# The Role of Obesity in the Etiology and Carcinogenesis of Endometrial Cancer

**DOI:** 10.7759/cureus.59219

**Published:** 2024-04-28

**Authors:** Alina-Gabriela Marin, Alexandru Filipescu, Aida Petca

**Affiliations:** 1 Obstetrics and Gynaecology, Elias Emergency University Hospital, Bucharest, ROU; 2 Obstetrics and Gynaecology, Carol Davila University of Medicine and Pharmacy, Bucharest, ROU

**Keywords:** risk factors, insulin resistance, inflammation, estrogen, endometrial cancer, obesity

## Abstract

Endometrial cancer, the most common gynecological malignancy, presents a complex public health challenge. While its incidence rises alongside the obesity epidemic, a well-established risk factor for endometrial cancer development, the impact of obesity on survival after diagnosis remains unclear. This review aims to explore the complex relationship between obesity and endometrial cancer’s development and survival rates, examining evidence from both epidemiological and clinical studies. It also aims to explore the proposed biological mechanisms by which excess adipose tissue promotes carcinogenesis and contributes to endometrial cancer progression and its negative effects on treatment outcomes. Furthermore, we analyzed the impact of body mass index, inflammation, hormonal imbalances, and their potential effects on endometrial cancer survival rates.

## Introduction and background

Endometrial cancer (EC), predominantly the endometrioid adenocarcinoma (EEC) phenotype, constitutes the most frequently diagnosed gynecological malignancy in high- and middle-income countries. A 2020 Globocan analysis revealed a number of 417,367 new cases, representing 2.2% of all new cancer diagnoses worldwide that year. However, data indicates that it accounted for 1.0% of global cancer deaths (97,370 new deaths), highlighting a lower mortality rate compared to other malignancies [1]. Intriguingly, both the incidence and mortality burden attributable to EC demonstrate substantial heterogeneity across socioeconomic development strata and geographic regions [1, 2].

The Bokhman dualistic model, based on an extensive clinicopathological analysis, categorizes endometrial cancer into two primary etiological groups [3]. Type I tumors, termed EECs, exhibit indolent behavior and a demonstrable association with hormonal imbalances. Epidemiological risk factors for the development of EEC are centered around unopposed estrogen (E) exposure without the counterbalancing effect of progesterone (P). These factors include obesity (body mass index (BMI) > 30 kg/m²), polycystic ovary syndrome (PCOS), nulliparity, early menarche, late menopause, and postmenopausal hormone therapy. These conditions are thought to contribute to an unopposed E-signaling state during perimenopause, potentially acting as a significant factor in neoplastic transformation within the female reproductive tract. Fortunately, most EECs are low-grade neoplasms (G1 or G2) with a glandular architecture and marked positivity for E receptor α (ERα). This favorable presentation facilitates early detection and timely intervention, resulting in excellent five-year survival rates exceeding 90% [4]. In addition, in type I EC, mutations in the tumor suppressor phosphatase and tensin homolog (PTEN) gene are the most prevalent, followed by K-ras mutations, β-catenin, PIK3CA mutations, PIK3 pathway alterations, and microsatellite instability (MSI) [5].

EC type II presents a contrasting clinical profile, characterized by aggressive behavior. These high-grade tumors arise from atrophic endometrium and demonstrate autonomy from E signaling, typically presenting in postmenopausal women [6]. In addition, they are characterized by a high frequency of p53 and p16 mutations, with concomitant downregulation of E-cadherin and HER2 amplification [5, 7]. Notably, despite the aggressive nature of type II EC, hormone-driven type I EC accounts for a higher number of deaths due to its higher incidence [8].

## Review

The global burden of obesity

Over the past three decades, the prevalence of obesity has witnessed a dramatic rise, impacting individuals across all ages, races, ethnicities, socioeconomic backgrounds, and geographical locations. Data from the World Health Organization (WHO) indicate that between 1975 and 2016, the number of overweight and obese adults has nearly tripled. In 2016, over 1.9 billion adults aged 18 years and older were classified as overweight by the WHO, with over 650 million categorized as obese. This translates to roughly 13% of the global adult population being obese in 2016, with a higher prevalence observed among women (15%) compared to men (11%) [9]. Regarding the Romanian population during the year 2016, data suggest a 25% obesity prevalence in adults, with a near-equal distribution among males (25%) and females (24%) [10].

Moreover, even childhood excess weight constitutes a significant and rising global public health concern, exhibiting increasing prevalence across diverse socioeconomic backgrounds. Overweight and obesity rates are demonstrably increasing, with a concomitant decrease in the prevalence of normal weight. This suggests a potential population-level shift toward higher weight categories, while underweight rates remain relatively stable [11]. The COVID-19 pandemic likely exacerbated this issue, as evidenced by multiple reports [12-14]. Current data highlight a concerning prevalence of 19.7% of childhood obesity in the United States [15]. Similarly, the European Region reports that one-third of children fall within the overweight or obese categories [16]. Romania mirrors this global trend of escalating childhood excess weight prevalence. Data from 2018 indicate a concerning prevalence of 18.5% of pediatric obesity, reflecting a significant increase from the 10% reported in 2010 [17, 18]. This highlights the urgency of addressing this public health issue.

**Figure 1:**
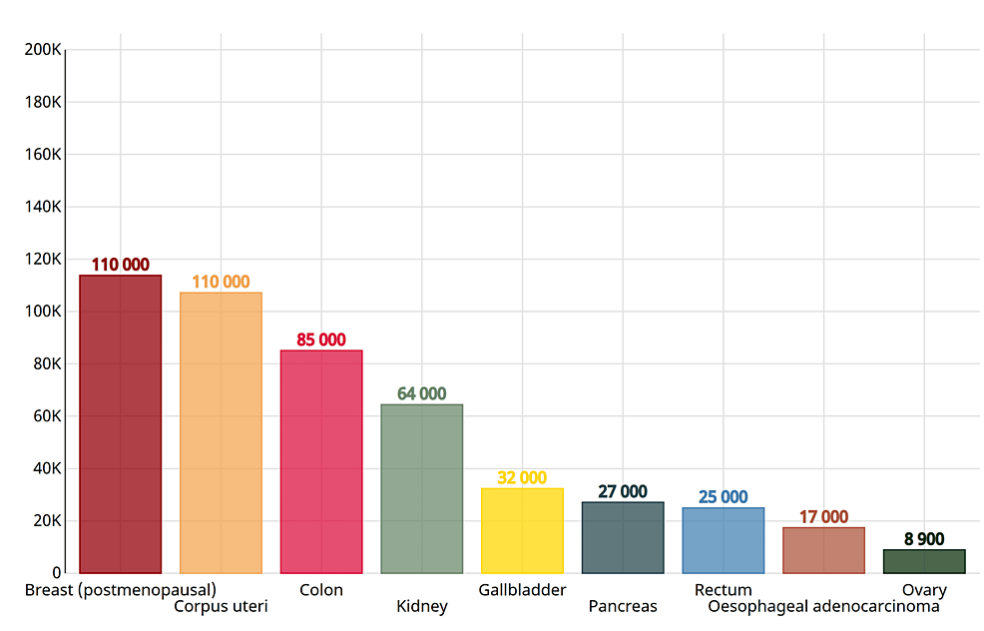
The number of new cancer cases in the United States in 2012 attributable to excess BMI: breast (postmenopausal), corpus uteri, colon, kidney, etc. (Globocan source: https://gco.iarc.fr/causes/obesity/tools-bars)

In Figure 1, Globocan analysis estimated the number of new cancer cases in the United States in 2012 attributable to excess body mass index (BMI) across various anatomical sites. Among the sites depicted, the figure suggests that excess BMI was associated with the greatest number of new cancer cases in the following organs: breast (postmenopausal), corpus uteri, colon, kidney, etc. (Globocan source: https://gco.iarc.fr/causes/obesity/tools-bars) [19].

Obesity and endometrial cancer: a dose-dependent relationship

Obesity is a well-established risk factor for EC. Existing literature reports a threefold increased risk in obese women compared to their normal-weight counterparts [20]. This association is further corroborated by an extended umbrella review demonstrating a robust positive correlation between BMI and EC incidence in both premenopausal (relative risk (RR) per 5 kg/m² = 1.49) and postmenopausal women (RR per 5 kg/m² = 1.60) [21].

An extensive meta-analysis involving a substantial sample size of 32,281,242 participants (20 prospective cohort and 20 case-control designs) solidified this connection. The analysis revealed a statistically significant, dose-dependent relationship between BMI and EC risk, with progressively increasing EC estimated RR and odds ratio (OR) observed across overweight (RR of 1.34 (95% confidence interval (CI): 1.20-1.48) and OR of 1.43 (95% CI: 1.30-1.56)), and obese (RR of 2.54 (95% CI: 2.27-2.81) and OR of 3.33 (95% CI: 2.87-3.79)) BMI categories compared to normal-weight individuals [22]. Notably, the ASTEC trial, a large international study based on the value of lymphadenectomy in early-stage disease, found that over 80% of EEC occurred in patients with BMI above 25 kg/m², with a further increase up to 50% in those exceeding BMI 30 kg/m² [23].

The association between obesity and EC extends beyond just the risk of developing the disease. A broad meta-analysis evaluating 46 studies investigated the relationship between obesity and clinical outcomes in women diagnosed with EC. The analysis revealed a statistically significant association between obesity and both overall mortality and cancer recurrence. Women with obesity exhibited a 34% increased risk of all-cause mortality (hazard ratio (HR) 1.34, 95% CI: 1.12-1.59) and a 28% increased risk of cancer recurrence (HR 1.28, 95% CI: 1.06-1.56) compared to their non-obese counterparts [24].

Recent US research analyzed data from over 540 women followed for a median of 14.2 years. It examined the longitudinal association between adiposity measurements, assessed both before and after the patients had been diagnosed with EC, as well as survival outcomes in these patients, and observed that both higher pre- and post-diagnosis BMI and waist circumference were significantly associated with poorer outcomes. Specifically, these measurements were linked to a decreased disease-free survival (DFS) and overall survival (OS) in the case of women who experienced weight gain in the year preceding diagnosis (peri-diagnostic) compared to normal or stable weight [25].

These findings highlight the significant impact of obesity on both the risk and prognosis of EC. Maintaining a healthy weight or losing weight after diagnosis may be crucial for improving clinical outcomes in women with this type of cancer.

Mechanisms linking obesity to endometrial cancer

Unveiling the Complex Hormonal Landscape of Endometrial Cancer

**Figure 2:**
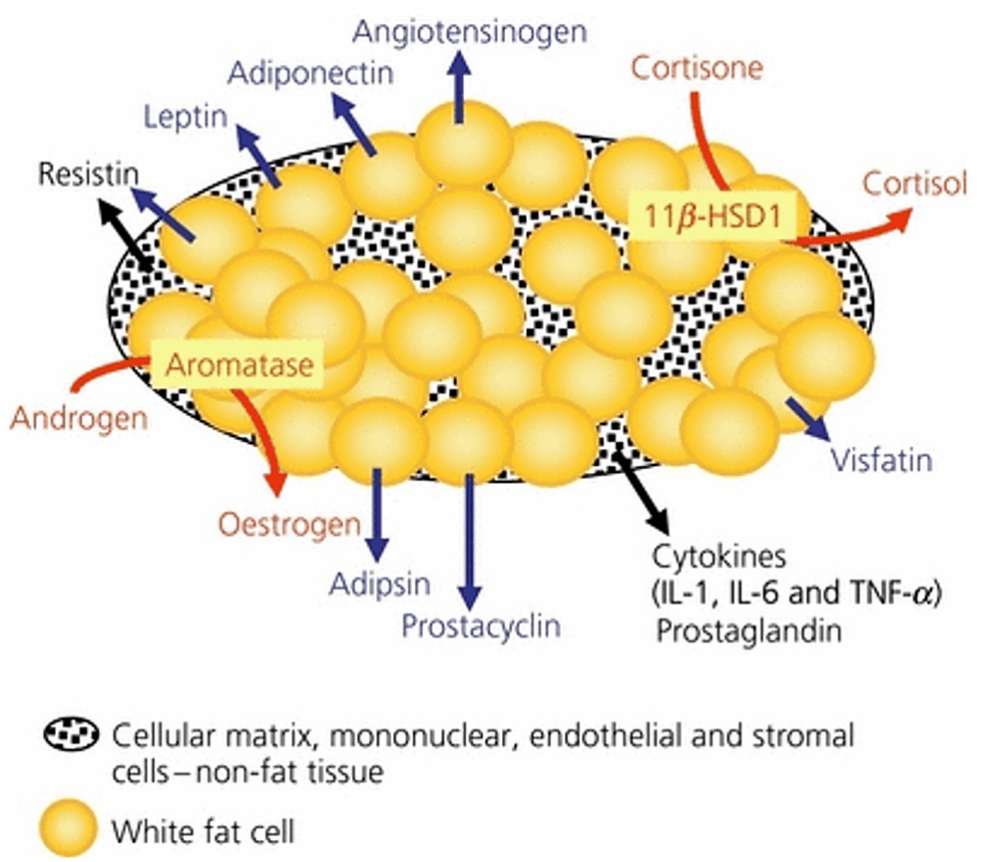
Adipose tissue hormones The figure highlights the endocrine role of adipose tissue through the ability of adipocytes to synthesize leptin, adiponectin, angiotensinogen, visfatin, prostacyclin, adipsin, and resistin (in rodents). Furthermore, the role of 11β-hydroxysteroid dehydrogenase 1 (11β-HSD1) and aromatase in converting inactive cortisone to the active glucocorticoid corticosterone (in rodents) or cortisol (in sheep and humans), and androgens to estrogens is emphasized. While once attributed solely to adipocytes themselves, recent research suggests the synthesis of interleukins (IL-1 and IL-6), tumor necrosis factor-alpha (TNF-α), and prostaglandins originates from cells within the adipose tissue matrix. These include mononuclear cells, endothelial cells, and stromal cells. The figure was reproduced with the permission of Iain J. Clarke B [28].

Obesity is hypothesized to contribute to endometrial carcinogenesis through several well-defined mechanisms that are resumed in Figure 2.

Estrogen Upregulation in Postmenopausal Women

One postulated mechanism involves the upregulation of E biosynthesis. In premenopausal women, cyclic ovarian estrogen production stimulates healthy endometrial proliferation during a normal menstrual cycle. However, after menopause, ovarian estrogen synthesis diminishes significantly. Moreover, adipose tissue, particularly in the postmenopausal state, acts as an endocrine organ and expresses significant levels of aromatase, an enzyme critical for the conversion of androgenic precursors, such as androstenedione and testosterone, to estrogens, including estradiol (E2) (see Figure 2). This enhanced enzymatic activity can lead to chronically elevated, obesity-fueled concentrations of unopposed estrogen that promote uncontrolled cell proliferation, a hallmark of neoplasia development [26-28].

Studies investigating the link between E levels and EC risk reveal a complex interplay between age and E type. A US study compared 208 postmenopausal women diagnosed with EC with 209 healthy controls. While high estrone levels were found to be associated with an increased EC risk (OR = 3.8, 95% CI: 2.2-6.6), this association weakened after adjusting for other factors, particularly BMI (OR = 2.2, 95% CI: 1.2-4.4). This suggests that obesity, a major source of estrone production after menopause, might be a stronger driver of EC risk than estrone itself. Similarly, elevated albumin-bound E2, a marker of bioavailable E, remained a significant risk factor even after adjustment (OR = 2.0, 95% CI: 1.0-3.9), suggesting its independent role [29]. In contrast to postmenopausal women, high concentrations of total, free, and albumin-bound E2 were not associated with an increased risk of EC in the premenopausal group [29].

Further supporting this age-related effect, a Norwegian study performed on 100 postmenopausal EC-diagnosed patients noted a significant positive correlation (p < 0.001) between circulating estrone and E2 levels and total abdominal fat (both visceral and subcutaneous compartments), but not specifically with visceral fat content [30]. This suggests that overall adipose stores, rather than just visceral fat, contribute to E production and potentially EC risk only after menopause.

E exerts its oncogenic effects on the endometrium through specific receptors that act as cellular messengers: E receptor alpha (ERα) and beta (ERβ). These receptors transmit signals to the cell nucleus, influencing gene expression. Studies utilizing established EC cell lines, such as Ishikawa and KLE, indicate that ERα and ERβ promote cellular processes crucial for tumor progression, such as invasion, migration, and proliferation. This suggests a potential link between receptor overexpression and aggressive EC behavior [31]. Further intrigue into E-mediated EC development comes from Chinese research that identified a novel player in E-mediated EC development: prohibitin, a protein whose expression is upregulated by E. This upregulation, mediated by ERα, might contribute to EC development by inhibiting protein degradation pathways [32].

The Influence of SHBG and Its Impact on “Free” Estrogen Levels

**Figure 3:**
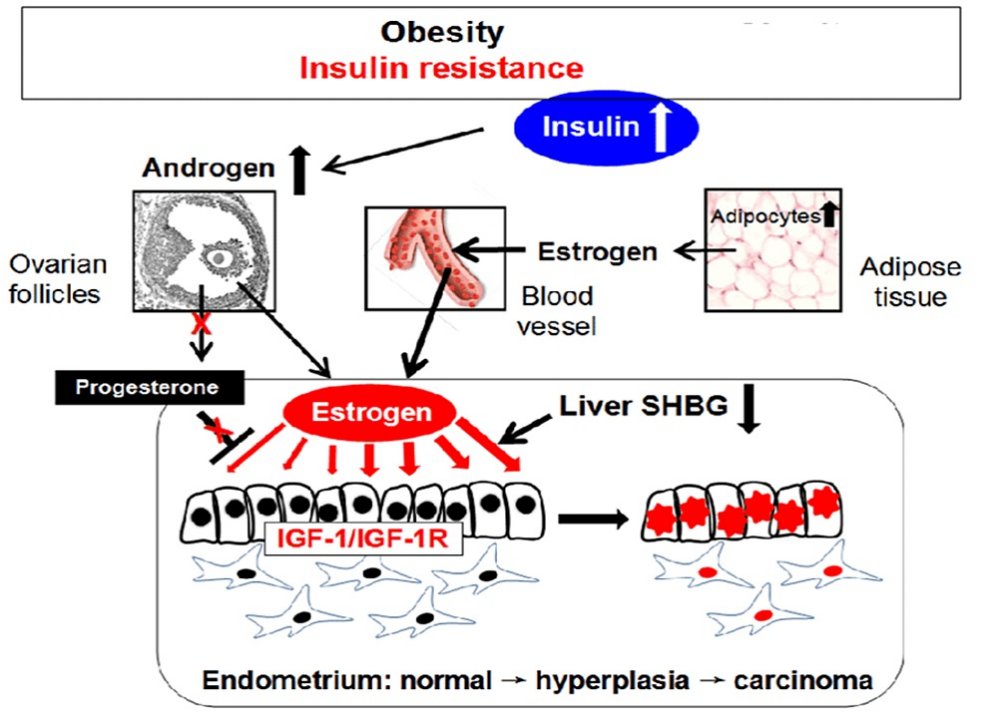
Endocrine impairment due to obesity promotes endometrial cancer development Evidence from clinical trials explains hormonal interactions between insulin resistance and obesity. Insulin resistance in these conditions stimulates estrogen production in the endometrium, leading to increased estrogen receptor activity. Additionally, activation of the IGF-1/IGF-1R pathway in epithelial cells further promotes endometrial cancer development. IGF-1, insulin-like growth factor-1; IGF- 1R, insulin-like growth factor-1 receptor; SHBG, sex hormone binding globulin; ↑, increase; ↓, decrease. The figure was reproduced with the permission of Linus Shao [35].

This rise in postmenopausal E production coincides with a decrease in sex hormone-binding globulin (SHBG), a protein responsible for transporting and regulating circulating E. Consequently, "free" or "bioactive" E levels increase, thus creating a hormonal milieu conducive to EC development. Indeed, studies have demonstrated a direct association between high unopposed "free" E and low SHBG levels with a heightened risk of EC. Conversely, high levels of SHBG in postmenopausal women were associated with a reduced risk of EC (OR = 0.51, 95% CI: 0.27-0.95), the association remaining significant even after adjusting for obesity [29]. Additionally, normal-weight or underweight women classified on the highest quartile of serum SHBG were less likely to develop EC, the risk being reduced by 61% (OR 0.39, p=0.02) when compared to women in the lowest quartile [33]. These findings highlight the protective role of SHBG in regulating E bioavailability and potentially reducing EC risk (see Figure 3) [34, 35].

Nonetheless, the Norwegian study suggests a more complex picture. This study investigated steroid hormone levels beyond E in 100 postmenopausal women diagnosed with EC. A significant association was observed between low serum levels of 17OH-progesterone, 11-deoxycortisol, and androstenedione and both aggressive tumor characteristics and poorer disease-specific survival (p-values of 0.003, 0.001, and 0.02, respectively). It was also noted that low levels of 17OH-progesterone and 11-deoxycortisol remained independent predictors of adverse outcomes (HR 2.69, p = 0.033, and HR 3.40, p = 0.02, respectively) even after adjusting for pre-operative risk factors like histological type and grade. Furthermore, patients with low steroid levels exhibited upregulated expression of genes associated with mitosis and cell cycle progression, as opposed to high steroid levels that were found to be correlated with increased activity in estrogenic signaling pathways and inflammatory genes [30].

Additionally, Canadian research compared 533 women diagnosed with EC with 976 controls and observed that an increased risk of EC was associated with elevated serum levels of androstenedione (OR 1.44, 95% CI 1.04-2.02) [33]. US research also observed a significant positive association between high circulating androstenedione levels and EC in both premenopausal (OR = 3.6, P for trend = 0.01) and postmenopausal women (OR = 2.8, P for trend < 0.001) [29].

While E signaling undeniably promotes EC development through various mechanisms, including DNA damage, enhanced angiogenesis, and increased cell proliferation and mutation, research suggests a more complex etiology, indicating that elevated E levels only partially explain the link between obesity and EC risk. BMI remains a significant independent factor [29].

Unveiling the Hidden Player: Chronic Inflammation and Endometrial Cancer

A second postulated mechanism involves chronic inflammation, a complex process arising from cellular dysfunction. German researchers have shed light on the potential roles of insulin resistance, metabolic syndrome, and inflammation in increasing EC risk among postmenopausal women. Their findings suggest a multifaceted interplay between hormonal and metabolic factors, highlighting the potential importance of inflammation beyond hormonal imbalances in EC development [36].

Inflammation's Yin and Yang: Macrophage Polarization

**Figure 4:**
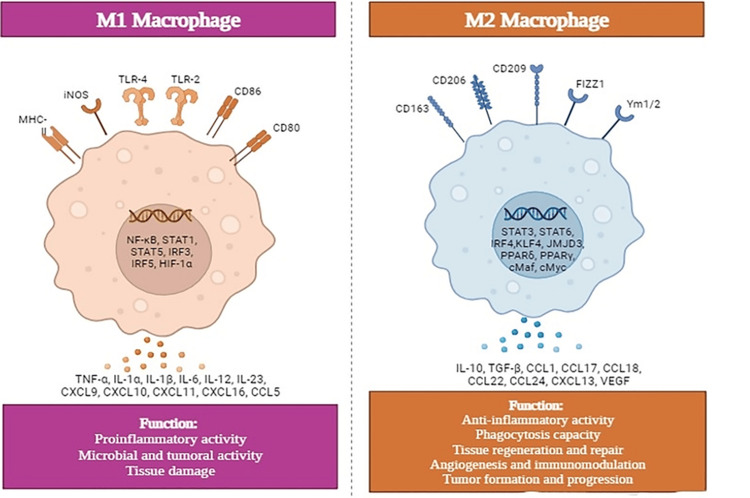
Macrophages subtypes: M1 and M2 Macrophages, crucial components of the immune system, are divided into two primary subtypes: M1 and M2. M1 macrophages (Pro-inflammatory) are stimulated by interferon-gamma (IFN-γ) and lipopolysaccharides (LPS), releasing pro-inflammatory cytokines (IL-1β, IL-6, TNF-α) to combat intracellular pathogens. They express high levels of CD86 and MHC II, essential for antigen presentation and T lymphocyte activation, and derive their energy from glycolysis, leading to significant reactive oxidative species (ROS) production. M2 macrophages (Anti-inflammatory), activated by IL-4, IL-13, and IL-10, promote the resolution of inflammation and facilitate tissue repair. They express CD163 and CD206 and rely on oxidative phosphorylation for their energy needs, resulting in lower ROS production. Maintaining a healthy balance between M1 and M2 macrophages is critical for tissue integrity. Any imbalance can lead to various diseases. Therefore, gaining a deeper understanding of the mechanisms that regulate the switch between M1 and M2 could pave the way for developing therapies that target macrophages. CCL, chemokine (C-C motif) ligand; cMaf, c-musculoaponeurotic fibrosarcoma; CXCL, chemokine (C-X-C) ligand; FIZZ1, resistin-like α; HIF, hypoxia-inducible factor; iNOS, inducible nitric oxide synthase; IFN-γ, interferon-gamma; IL, interleukin; IRF, interferon regulatory factor; JMJD, Jumonji domain-containing protein; KLF, Kruppel-like factor; NF-κB, nuclear factor κB; KLF, Kruppel-like factor; LPS, lipopolysaccharides; MHC, major histocompatibility complex; PPAR, peroxisome proliferator-activated receptors; STAT, signal transducer and activator of transcription; TLR, Toll-like receptor; TNF-α, tumor necrosis factor alpha; TGF-β, transforming growth factor beta; VEGF, vascular endothelial growth factor; Ym1, chitinase 3-like 3. The figure was reproduced with permission from Xiang-Hong Xu [40].

Obesity-induced adipose tissue dysfunction is associated with chronic, low-grade inflammation, distinguished from transient acute inflammation. This condition manifests as a persistent state characterized by the infiltration of adipose tissue with lymphocytes and macrophages, the latter becoming polarized toward a pro-inflammatory M1 phenotype (see Figure 4), further amplifying the inflammatory response [37-40]. Chronic inflammation has also been linked to compromised blood flow observed in expanding adipose tissue, a factor that leads to localized hypoxia. This oxygen deprivation activates pro-inflammatory signaling pathways within adipocytes, promoting tissue necrosis and further macrophage infiltration [41-44]. Moreover, overloaded adipocytes and the associated mechanical stress can directly activate pathogen-recognition receptors on immune cells, bypassing the need for external stimuli and promoting chronic inflammation [45].

The key mechanisms of the obesity-linked pro-inflammatory state also consist of excessive nutrient intake leading to a cellular stress response pathway through the accumulation of misfolded proteins within the endoplasmic reticulum, which then induces the expression of pro-inflammatory cytokines [46-51].

Additionally, free fatty acids released from hypertrophic adipocytes can bind to Toll-like receptors (TLRs) on the surface of immune cells, particularly TLR4 and TLR2. This interaction activates downstream signaling pathways such as Nuclear Factor Kappa B (NF-κB) and c-Jun N-terminal kinase 1 (JNK1), leading to the production of chemokines that recruit additional pro-inflammatory macrophages, perpetuating the inflammatory cascade [52-55].

From Biomarkers to Risk Assessment

Oncogenesis and tumor progression are often accompanied by the release of inflammatory mediators into the bloodstream. These mediators, generally known as inflammatory biomarkers-CRP, IL-6, and TNFα-can serve as valuable indicators of underlying pathologies, including endometrial cancer (EC). CRP, an acute phase protein, is a sensitive marker of systemic inflammation linked to various afflictions including diabetes mellitus (DM) [56], cardiovascular disease [57], and overall cancer risk (including breast cancer, melanoma, and cervical and colon cancer) [58]. Studies have shown a significant correlation between elevated CRP levels (>10 mg/L vs ≤1 mg/L) and increased risk of epithelial ovarian cancer [59-61], and in some cases, it is marked as an independent prognostic factor for ovarian cancer [62]. Additionally, CRP was significantly linked to persistent or recurrent papillary thyroid carcinoma [63]. Several studies have established a link between CRP and EC risk, particularly type I EC. This association appears to be stronger with increasing BMI, suggesting that CRP might not be an independent risk factor but may increase the risk in the presence of obesity, particularly central obesity [64]. The presence of DM in EC patients further contributes to chronic inflammation and increased oxidative stress [64]. CRP gene polymorphisms might also play a role [65].

While CRP seems to hold promise, the picture is less clear for other inflammatory markers. A meta-analysis conducted in the UK that included 18 articles involving 2,921 women diagnosed with EC and 5,302 controls revealed no significant correlations between the highest and lowest TNFα or IL-6 levels and the risk of EC [66]. Measuring CRP levels could be valuable for EC diagnosis and prognosis. Elevated CRP has been linked to both an increased risk of developing EC and a poorer prognosis for affected patients [67].

Obesity, Inflammation, and Insulin Resistance: A Triple Threat for Endometrial Cancer

A third postulated mechanism is that of insulin resistance and its association with metabolic syndrome. Obesity, often a component of metabolic syndrome, includes a cluster of conditions such as high blood pressure, abnormal cholesterol levels, and high serum glucose levels. Obesity is frequently accompanied by insulin resistance, where cells become less responsive to insulin. Additionally, insulin has growth-promoting effects besides its role in regulating glucose levels.

These factors may independently or synergistically contribute to EC risk, particularly through associated chronic inflammation characterized by dysregulated cytokine production, elevated acute-phase reactants, and activation of inflammatory signaling pathways [68]. Regarding pro-inflammatory reactants, TNF-α was first found to be overexpressed in adipose tissue from obese mice [69], and later in adipose and muscle tissues from obese humans [70]. These findings both impede insulin action and induce insulin resistance, particularly in the case of exogenous TNF-α administration. Moreover, TNF-α dysfunction was shown to improve insulin sensitivity and glucose homeostasis, solidifying the critical role of this inflammatory response in regulating insulin action during obesity [71, 72]. Improvements in systemic insulin sensitivity in humans treated with anti-TNF-α therapies for inflammatory diseases like rheumatoid arthritis also suggest a potential therapeutic role for TNF-α [73].

Obesity is linked to impaired insulin signaling, partly due to inflammatory signals generated within adipose tissue. Insulin resistance arises in part due to dysfunctional insulin signaling. Unlike other receptors of the tyrosine kinase family, insulin and IGF receptors rely on docking proteins to transmit signals. Within the cell, insulin activates insulin receptor substrate (IRS) proteins (IRS-1 to -6) through tyrosine phosphorylation, a crucial step that is disrupted in most cases of insulin resistance. TNF-α further hinders this process by promoting inhibitory serine phosphorylation of IRS-1. This modification, observed in insulin-resistant cells and tissues, blocks the transmission of insulin’s message within the cell. Kinases like JNK and an inhibitor of nuclear factor kappa-B kinase beta (IKKβ) are culprits in this disruptive process. Notably, JNK activity is high in obese tissues, and it has been observed that mice lacking JNK1 and IKKβ activity are protected from insulin resistance. These findings position JNK and IKKβ as potential targets for treating insulin resistance [74, 75].

The observed correlation between the presence of pro-inflammatory cytokines, the recruitment of myeloid cells, and the metabolic dysfunction seen in obese patients is well-established. Notably, obesity-induced insulin resistance further disrupts the delicate balance of the adaptive immune response. Studies utilizing diet-induced obesity models in mice have demonstrated impaired T cell function, consequently leading to increased susceptibility to viral infections such as influenza [76, 77].

Future directions

The link between obesity and EC suggests promising avenues for future research and prevention. The strong correlation between BMI and EC risk highlights the potential of weight management programs designed specifically for high-risk populations, with tailored strategies for long-term adherence and maximizing their impact on EC prevention. Future research should also focus on deciphering the specific inflammatory pathways involved in the development of EC and explore potential anti-inflammatory therapies. Moreover, validating the role of prohibitin and exploring its potential as a therapeutic target for disrupting E-mediated EC development could offer new avenues for treatment and prevention strategies.

## Conclusions

Increasing BMI levels are directly correlated with a proportional increase in EC risk, underscoring the potential significance of weight management interventions in EC prevention strategies. Obesity triggers chronic inflammation through a multifactorial process involving ER stress, macrophage infiltration and polarization, hypoxia-induced inflammatory signaling, direct immune system activation, and free fatty acid-TLR signaling. This chronic inflammatory state contributes to the development of various obesity-associated comorbidities. Moreover, obesity modulates the production and bioavailability of endogenous sex steroids, leading to chronic hyperinsulinemia and activation of cellular growth factor signaling pathways, ultimately promoting tumorigenesis.
